# Multi-segment cooling design of a reflection mirror based on the finite-element method

**DOI:** 10.1107/S1600577524009664

**Published:** 2025-01-01

**Authors:** Zhen Wang, Yajun Tong, Fang Liu, Chaofan Xue, Limin Jin, Zhi Liu

**Affiliations:** a Center for Transformative Science ShanghaiTech University 393 Middle Huaxia Road Shanghai201210 People’s Republic of China; b Shanghai Institute of Applied Physics Chinese Academy of Sciences Shanghai201204 People’s Republic of China; Tohoku University, Japan

**Keywords:** finite-element analysis, high heat load, height error, cooling optimization

## Abstract

High-repetition-rate free-electron lasers impose stringent requirements on thermal deformations of optics in the beamline. The Shanghai HIgh-repetition-rate XFEL aNd Extreme light facility (SHINE) experiences high average thermal power and demands wavefront preservation. To effectively manage thermal deformation in the first reflection mirrors M1, we optimized the cooling length and position of the cooling groove with numerical calculations. For example, the root mean square of the height error of the thermal deformation of the mirror at a photon energy of 900 eV was optimized, resulting in a 12.7× reduction, from 13.76 nm to 1.08 nm. This optimized design also eliminated stray light in the focus spot at the sample and resulted in a 177% increase in the peak intensity of the beam’s focus spot at the sample, from 3.08 × 10^5^ to 8.53 × 10^5^. The multi-segment cooling design of the mirror advanced the quality of the beam’s focus spot at the sample and ensured the stable operation of SHINE under high repetition rates.

## Abstract 2

Through numerical optimization of cooling lengths and cooling groove positions for the first reflection mirror of a free-electron laser, the root mean square of the height error of the mirror’s thermal deformation was minimized. The optimized mirror design effectively mitigated stray light and enhanced the peak intensity of the focus spot at the sample, thereby enhancing the optical performance of the high-heat-load mirror under high repetition rates at beamline FEL-II of the SHINE facility.

## 1. Introduction

The SHINE facility in Shanghai represents a significant advancement as it is China’s inaugural high-repetition-rate X-ray free-electron laser (XFEL) device. During its first phase, three beamlines were established — FEL-I, FEL-II and FEL-III — spanning a photon energy range from 0.4 to 25 keV. Notably, the FEL-II beamline operates within the 0.4–3 keV photon energy range and includes four endstations. As illustrated in Fig. 1 of Wang *et al.* (2024 ▸), the beam transport system extends approximately 348.6 m and links to four experimental stations: the Soft X-ray Scattering Spectrometer Endstation (SSS), the Spectrometer for Electronic Structure EndStation (SES), the Coherent Diffraction Endstation (CDE), and the Atomic, Molecular and Optical Physics Endstation (AMO) (Zhu *et al.*, 2017 ▸; Liu *et al.*, 2023 ▸; Qi *et al.*, 2022 ▸).

XFELs are scientific research tools known for their high brightness, high coherence and ultra-short pulses (Eriksson *et al.*, 2014 ▸; Hettel, 2014 ▸). They provide unmatched detection capabilities and have been applied across multiple research fields and various disciplines. The evolution of X-ray light sources has advanced from the initial X-ray tube to synchrotron light sources, ultimately leading to the current diffraction-limited storage rings and XFELs (Emma *et al.*, 2010 ▸; Ishikawa *et al.*, 2012 ▸; Kim *et al.*, 2017 ▸; Pellegrini *et al.*, 2016 ▸).

However, because of their brightness and coherence, there are strict requirements for transmitting X-rays, which include damage resistance, high stability and wavefront preservation (Cocco & Spiga, 2019 ▸; Church & Takacs, 1993 ▸). The new generation of high-repetition-rate XFELs has significantly increased the average power, which results in a high heat load on the beamline’s optical components.

XFEL beamlines experience high average power and demand wavefront preservation. The light source is very bright and has high energy density (Altarelli, 2015 ▸). However, the first reflection mirror M1 in the beamline has to withstand a high heat load, resulting in severe thermal deformation of the mirror, which could influence the focus spot imaging of X-rays at the sample in the endstation. Therefore, the thermal management of optical components under high heat load by Project SHINE is a key technical challenge.

Techniques to control the thermal deformation of optical components in synchrotron radiation and FEL facilities can be classified as active or passive. Active methods, such as the multi-segmented piezoelectric (PZT) approach at the European Synchrotron Radiation Facility (ESRF) and the European XFEL, are considered more advanced (Signorato *et al.*, 1998 ▸; Yang *et al.*, 2014 ▸; Cocco *et al.*, 2020 ▸; Yang *et al.*, 2020 ▸; Xu *et al.*, 2023 ▸). However, the control system of active methods is complex and still has some real-time measurement problems in practical applications (Sutter *et al.*, 2022 ▸). Passive methods, such as the local side cooling scheme at Shanghai Synchrotron Radiation Facility (SSRF) (Xu & Wang, 2012 ▸) and ESRF’s slotted mirror design (Zhang *et al.*, 2013 ▸; Zhang *et al.*, 2015 ▸), are easier to control and widely used in many beamlines.

This paper presents an improved design with combined optimization of the cooling length and position of the cooling groove in the mirror. The optimal design of the mirror was finally determined with the height-error value of the mirror’s thermal deformation and the results of optical tracing. The theory of heat transfer and the cooling scheme of mirrors are first introduced, then the finite-element model of thermal analysis is explained, and the mirror’s thermal deformation under high heat load was calculated. According to the resulting height error of the mirror’s thermal deformation, the design parameters of the mirror cooling schema are optimized. Finally, the optimized mirror design achieved the highest optical performance for the XFEL beamline.

## 2. Theory of thermal analysis

### 2.1. Classic theory of heat transfer

In the classic theory of heat transfer in a closed system, thermal analysis abides by the first law of thermodynamics (Joule *et al.*, 1843 ▸), the law of conservation of energy,
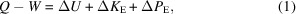
where *Q* indicates the heat (J), *W* means the work (J) and 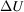 is the internal energy (J). Additionally, 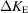 describes kinetic energy (J) and 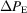 the potential energy (J).

Since the kinetic and potential energies of a solid heat transfer system in most engineering are ignored, equation (1) for steady-state thermal analysis develops as
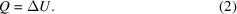
The heat flowing into the system equals the heat flowing out.

### 2.2. Heat conduction

Heat conduction in solids can be described as the transfer of internal energy resulting from a temperature gradient, occurring either between two objects in direct contact or among different parts of a single object. Heat conduction was described using Fourier’s law (Fourier *et al.*, 1822 ▸),
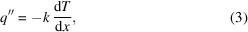
where 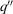 expresses the heat flow density (W m^−2^), *k* indicates the coefficient of thermal conductivity (W m^−1^ °C^−1^), and the minus sign indicates that the heat flows in the direction of decreasing temperature.

### 2.3. Thermal convection

Thermal convection refers to the exchange of heat caused by the temperature difference between the surface of a solid and the fluid in contact with it. It can be divided into two categories: natural convection and forced convection. Thermal convection is described by Newton’s law of cooling (Newton *et al.*, 1701 ▸),
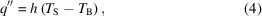
where *h* is defined as the convective heat transfer coefficient (W m^−2^ °C^−1^), *T*_S_ is the solid surface temperature (°C), and *T*_B_ is the surrounding fluid temperature (°C). Thermal analysis of the reflection mirror was executed based on the theory of heat conduction and thermal convection.

### 2.4. Cooling water convection heat transfer

The convective heat transfer coefficient on the inner wall of a cooling water tube is calculated using equation (9),
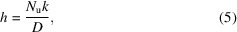
where *h* is the convective heat transfer coefficient (W m^−2^ °C^−1^), *N*_u_ is the Nusselt number, *k* is the thermal conductivity of the cooling medium (W m^−1^ °C^−1^) and *D* is the inner diameter of the circular cooling tube (mm).

According to the Dittus–Boelter formula (Dittus & Boelter, 1985 ▸),
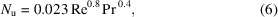

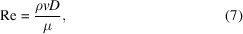

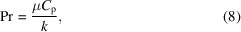

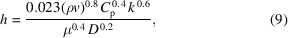
where Re is the Reynolds number, reflecting the effect of convective strength on heat transfer, Pr is the Prandtl number, reflecting the effect of fluid properties, ρ is the density of the cooling medium (kg m^−3^), *v* is the velocity of the cooling medium (m s^−1^), μ is the viscosity of the cooling medium (Pa s) and *C*_p_ is the specific heat of the cooling medium (J kg^−1^ °C^−1^). Note that the Dittus–Boelter equation is generally applicable to smooth-bore circular tubes where the fluid flow is in a vigorous turbulent state, with a Reynolds number Re greater than 10000, a Prandtl number Pr greater than 0.7, and *L*/*D* greater than 10, where *L* and *D* are the length and inner diameter of the circular cooling tube.

## 3. Finite-element method

Finite-element analysis was performed using the numerical finite software *Ansys Workbench.*

### 3.1. Geometry model

Based on the current optical design specifications for the FEL-II beam, the mirror M1 has been designed as shown in Fig. 2 of Wang *et al.* (2024 ▸). The mirror’s dimensions are 900 mm (*X*) × 60 mm (*Y*) × 60 mm (*Z*), and it is constructed from single-crystal silicon owing to its superior thermal conductivity and low thermal expansion coefficient. Addressing the considerable heat load challenge, the cooling blade for the mirror is designed and manufactured from oxygen-free copper to guarantee optimal heat transfer. The cooling blade tube has an inner diameter of 8 mm, which further enhances the heat dissipation capability of the mirror system. To maintain stability standards for the 3.1 km hard XFEL facility, it is advised to implement liquid indium–gallium (In–Ga) eutectic in the upper cooling groove of the mirror. This approach effectively isolates cooling blade vibrations while ensuring efficient heat transfer.

The properties of the applied materials for the thermal analysis are listed in Table 1 ▸ of Wang *et al.* (2024 ▸), based on the FEL-II beam design specifications for Project SHINE.

### 3.2. Heat load and boundary conditions

**Figure 1:**
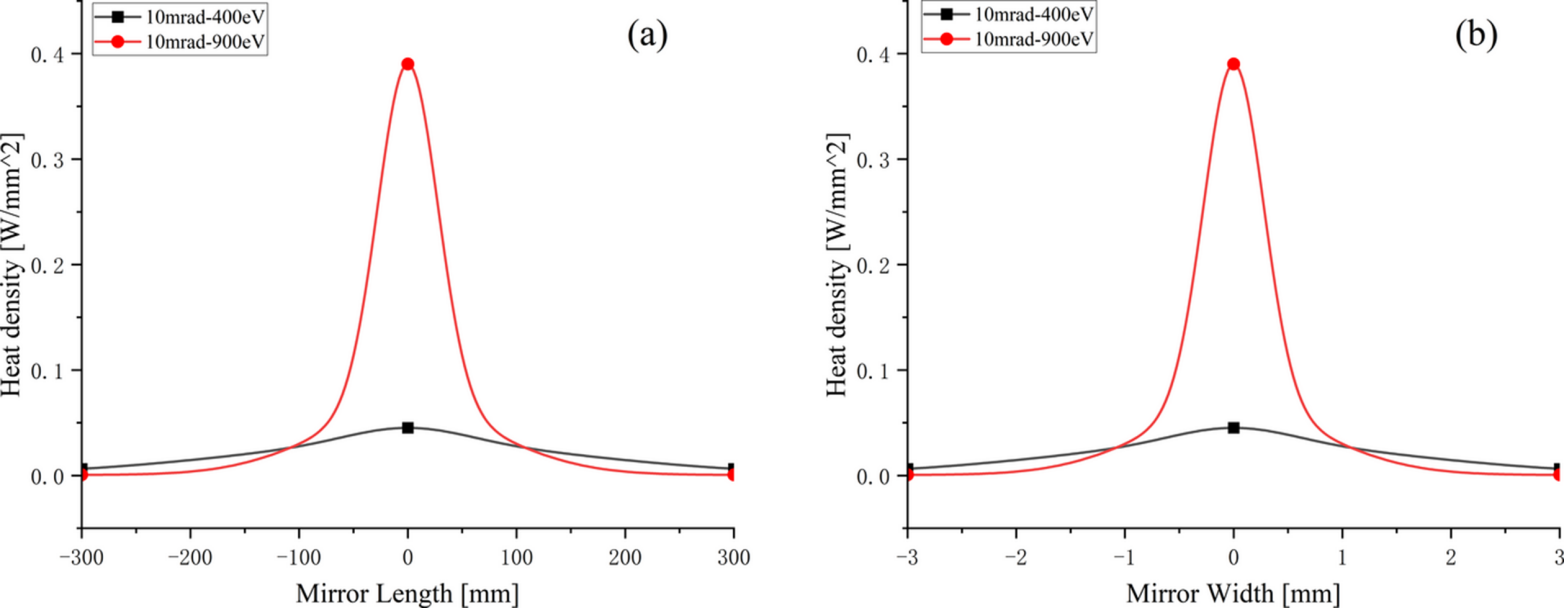
Heat load distribution of the footprint on reflection mirror M1.

The mirror was positioned 165 m from the light source, with glancing angles on the first reflection mirror’s optical surface set at 10 mrad for photon energies of 400 eV, 900 eV and 2000 eV, and 5 mrad for photon energies of 2000 eV and 3000 eV. The configuration with a glancing angle of 5 mrad is designed to reduce the thermal power density of the X-rays at higher photon energies of 2000 eV and 3000 eV on mirror M1. This study primarily focuses on the thermal analysis of the 400 eV and 900 eV photon energies. Fig. 1 ▸ illustrates the power density distribution for these photon energies. The heat load was concentrated in the center of the mirror’s optical surface, with a footprint size of 600 mm (*X*) × 6 mm (*Y*). Mirror M1 endured a maximum heat load of 43.3 W at a photon energy of 900 eV within a repetition rate of 1 MHz, leading to a peak power density of 0.38 W mm^−2^.

Furthermore, the coefficient of thermal contact conductance between the In–Ga alloy and the single-crystal silicon within the mirror system and cooling tube is reported to be 150000 W m^−2^ K^−1^ (Khounsary *et al.*, 1997 ▸). The cooling mechanism at the inner tube wall is characterized by a fixed convective heat transfer coefficient, which is associated with a temperature of 30°C and a cooling water flow rate of 1.5 L min^−1^. This equivalent convective heat transfer coefficient was calculated to be 3177 W mm^−2^ K^−1^, as derived from equation (9) and the material properties of the cooling water listed in Table 1 ▸.

### 3.3. Model of the finite-element method

**Figure 2:**
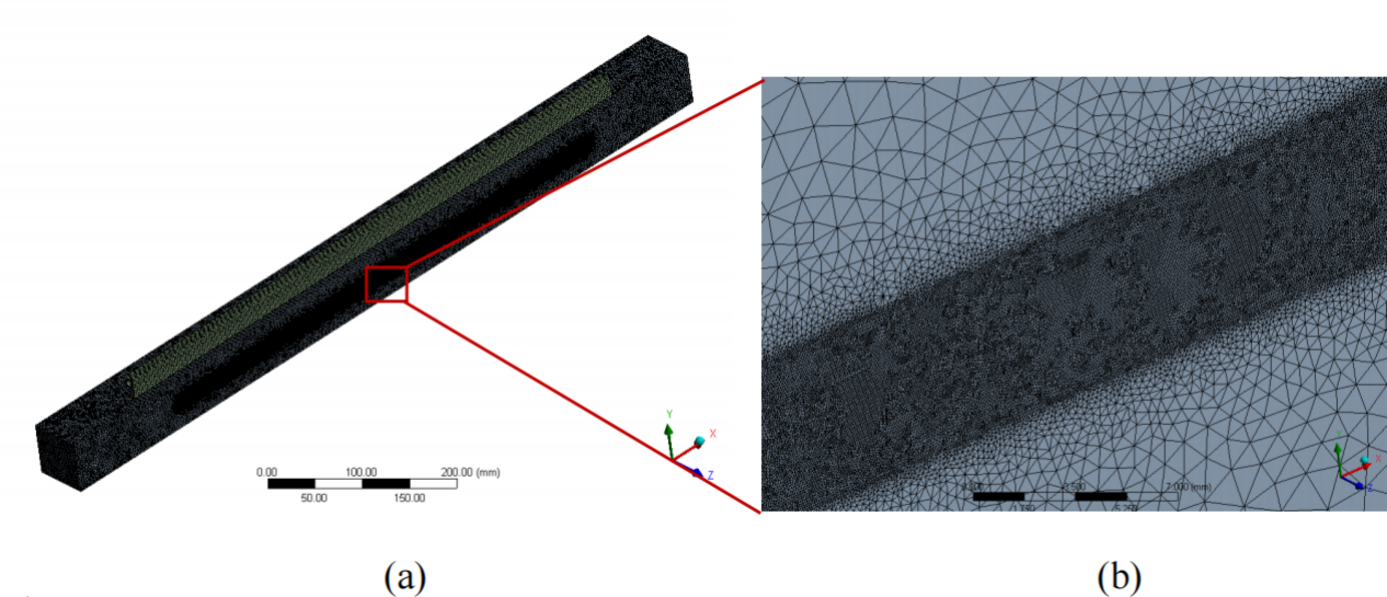
(*a*) Finite-element model of the mirror system. (*b*) Mesh of the footprint area.

The finite-element analysis model of the mirror system assembly, illustrated in Fig. 2 ▸, employed a global element size of 3 mm and utilized tetrahedral elements. To capture the Gaussian distribution characteristics of heat density in the high thermal density central region accurately, a local fine mesh was applied, with some areas having a footprint as small as 0.1 mm. The detailed model comprised 12096701 elements and 17169069 nodes.

## 4. Results and discussions

### 4.1. Full-length cooling schema design

The original mirror design featured an extensive cooling system, with the cooling channel positioned 10 mm from the optical surface and spanning a full 500 mm in length. According to the experimental station’s specifications for the focused beam spot, the thermal analysis for mirror M1 under a repetition rate of 330 kHz was performed, and the residual height error due to thermal deformation was evaluated using the best spherical fit (BSF) approach (Zhang *et al.*, 2015 ▸). The root mean square (RMS) deviation of this error was found to be 9.17 nm at a photon energy of 400 eV, which escalated to 13.76 nm at a higher photon energy of 900 eV.

**Figure 3:**
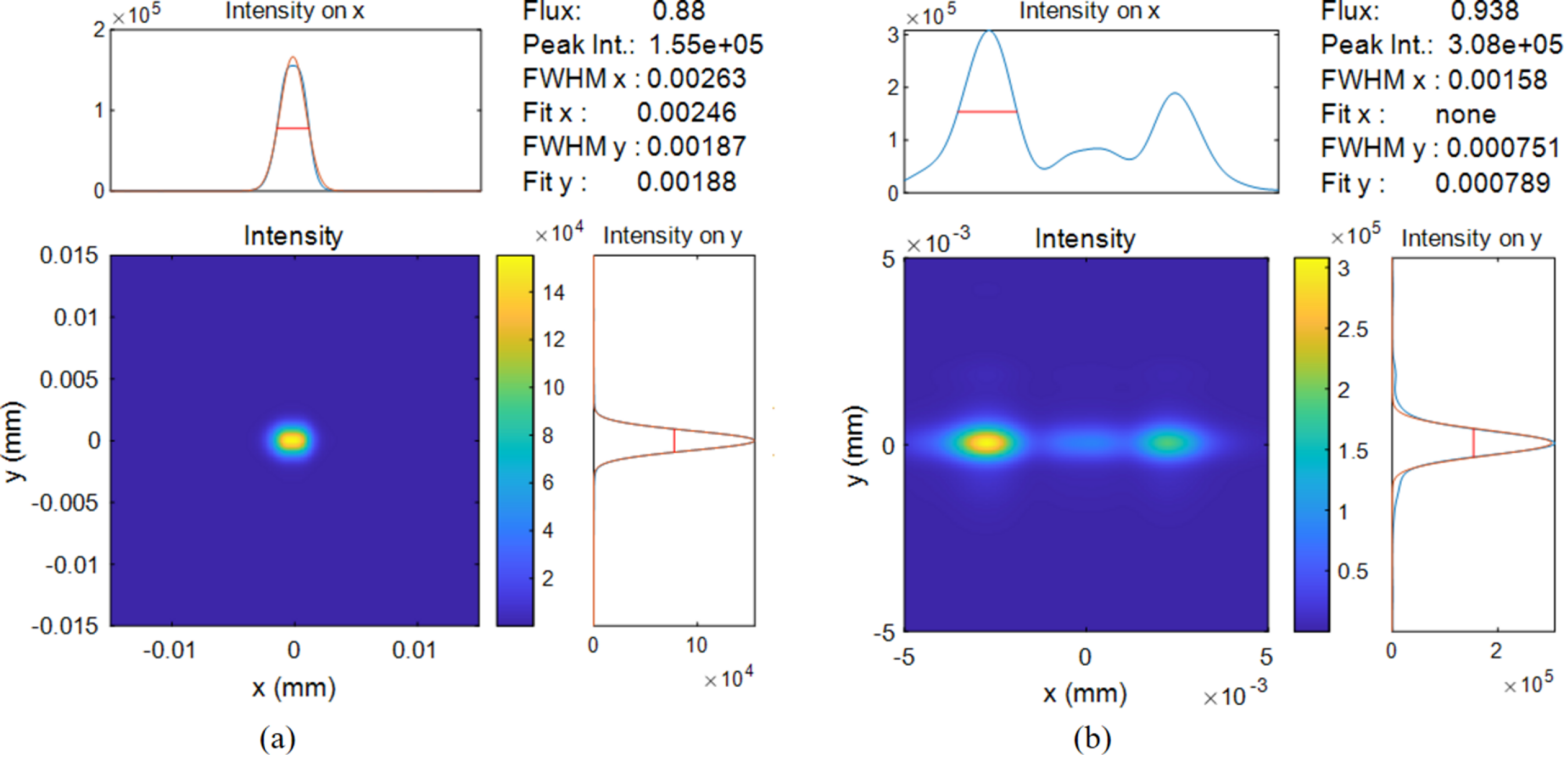
Focusing spot at the sample for the full-length cooling design for (*a*) 400 eV and (*b*) 900 eV.

Additionally, the impact of thermal deformation on the beam’s imaging performance at the sample point was examined using the *MOI* package developed by SSRF (Meng *et al.*, 2015 ▸; Meng *et al.*, 2017 ▸; Ren *et al.*, 2019 ▸). This package, which is based on statistical optics for the numerical analysis of partially coherent X-ray beams, has been successfully applied in the beamline design and analysis of coherent light propagation in both synchrotron (Xue *et al.*, 2018 ▸) and free-electron laser (Xue *et al.*, 2024 ▸) facilities. The resultant focus spot images of the X-ray beamline at the sample point for 400 eV and 900 eV photon energies are presented in Figs. 3 ▸(*a*) and 3 ▸(*b*), respectively. It was evident that there was stray light in the focus spot for the 900 eV photon energy image and the focus performance for the 400 eV photon energy image was deficient. Compared with the focusing effect for photon energy 400 eV, the beamline of photon energy 900 eV is poorer focused at the sample point, mainly due to the shorter full width at half-maximum (FWHM) of the 900 eV footprint and the higher peak value of thermal flux in the footprint, resulting in a more concentrated heat load on the mirror’s optical surface, which ultimately led to more severe thermal distortion on the optical surface of the mirror.

According to the optical tracked results of the mirror’s thermal deformation in full-length mirror design, the focus effect of the beamline at the sample point cannot meet the technical requirements of the experimental station. An optimization design of the full-length cooling mirror must be carried out to eliminate the stray light in the focused spot and to improve the focusing effect of the X-ray beamline at the sample point, to fulfill the experimental requirements of the experimental station.

### 4.2. Optimization of the cooling schema and mirror profile

**Figure 4:**
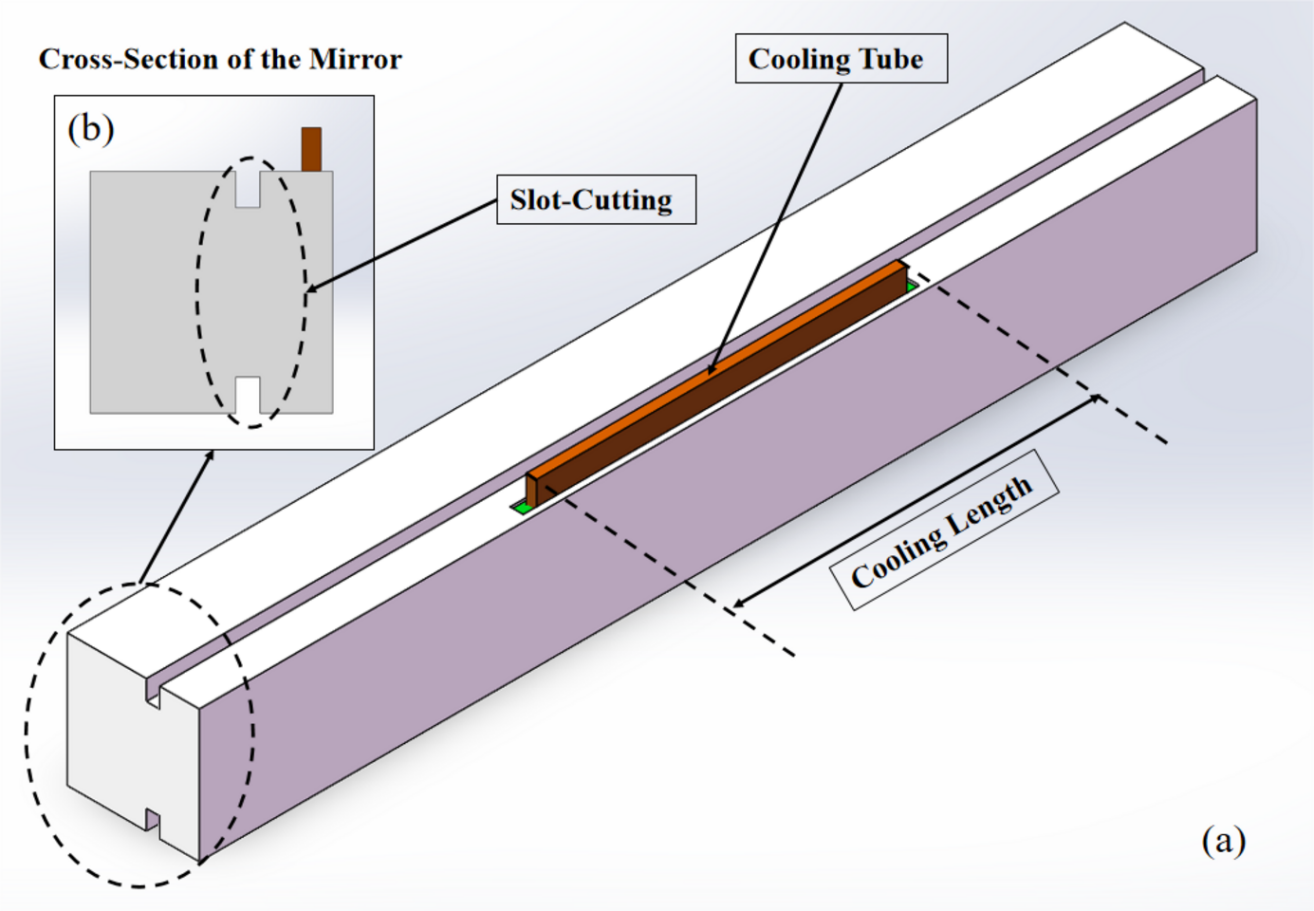
Optimized method of X-ray mirror thermal deformation. (*a*) Design of the X-ray mirror. (*b*) Cross section of the X-ray mirror.

By studying and researching the reference literature on the optimization design of X-ray mirror thermal deformation, it was realized that Zhang and co-workers had found in their research that modifying the cross section of the mirror by slot-cutting (Zhang *et al.*, 2013 ▸) and optimizing the cooling length of the cooling tube (Zhang *et al.*, 2015 ▸) significantly affected the thermal deformation of X-ray mirrors, as displayed in Figs. 4 ▸(*a*) and 4 ▸(*b*). In their research, the optimal cooling length of the cooling tube and a reasonable cross-section design of the mirror could achieve better management of the mirror’s thermal deformation.

Following the aforementioned investigation, the mirror design was optimized with a focus on the cooling length and the position of the cooling grooves. These parameters were identified as having the most pronounced and direct impact on the mirror’s thermal deformation and the quality of the focus spot at the sample location.

**Figure 5:**
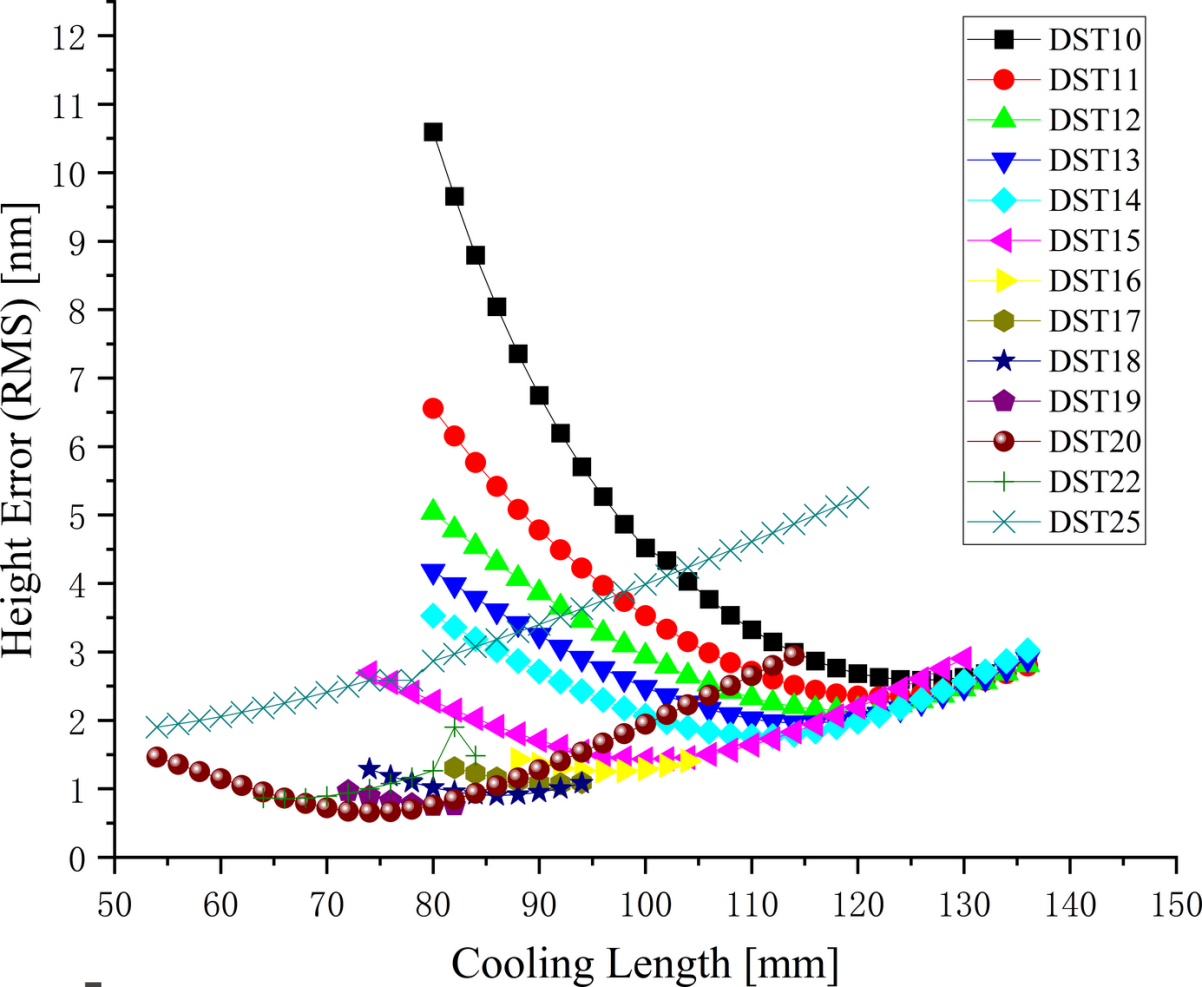
Height error in the optimization process.

Initially, the optimization efforts were focused on a photon energy of 900 eV, recognized as the most important energy point and concurrently the one with the poorest imaging effect of the beamline at the sample. In this study, the cooling length varied from 50 to 140 mm, while the position of the cooling groove (DST) was adjusted between 10 and 25 mm. As depicted in Fig. 5 ▸, the height error (RMS) obtained through the BSF approach was significantly influenced by the cooling length and the position of the cooling groove. For cooling groove positions within the range 10–20 mm there was an optimal cooling length at each fixed cooling groove position. Moreover, as the cooling groove position increased, the cooling length gradually decreased, and the height error (RMS) also diminished. However, when the cooling groove position exceeded the range of 20 mm, it became difficult to obtain an optimal solution for the cooling length and the height error (RMS). Additionally, in this optimization process, we also needed to consider the supporting structure design of the mirror, so the cooling groove position of the mirror was finally fixed with DST = 18 mm, and a cooling length of 90 mm was applied for the photon energy 900 eV. The height error (RMS) of the thermal deformation was reduced to 1.08 nm by this optimal mirror design.

Using the same method as previously described, the optimization for the cooling length at the 400 eV energy point was executed with the cooling groove positioned at 18 mm. Upon completion of the optimization process, it was found that the height error (RMS) achieved its lowest value of 1.55 nm at an optimized cooling length of 340 mm.

**Figure 6:**
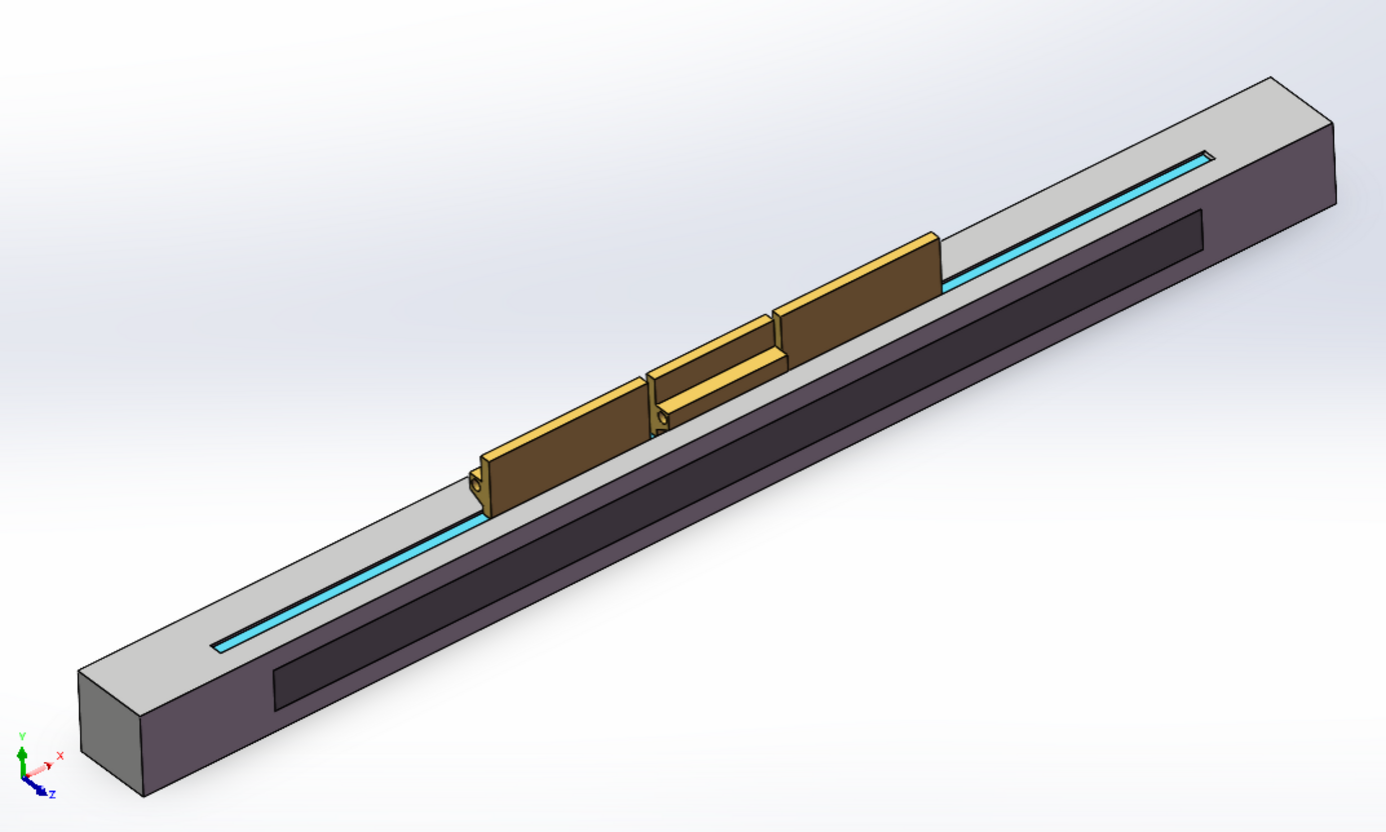
Multi-segment cooling design of the mirror. Cooling mode 1: activating the middle cooling tube. Cooling mode 2: activating the three-section cooling blade.

Finally, a multi-segment cooling design for mirror M1 of the FEL-II beamline was obtained, as displayed in Fig. 6 ▸. During the operation of the beamline at a photon energy of 900 eV, cooling mode 1 is switched on. This mode specifically activates the central cooling plate to dissipate the heat of the mirror, while the side cooling plates remain deactivated. Conversely, when the beamline is operated at a photon energy of 400 eV, an alternative cooling strategy, known as cooling mode 2, is deployed. This advanced mode concurrently activates all three cooling segments.

### 4.3. Discussion

**Figure 7:**
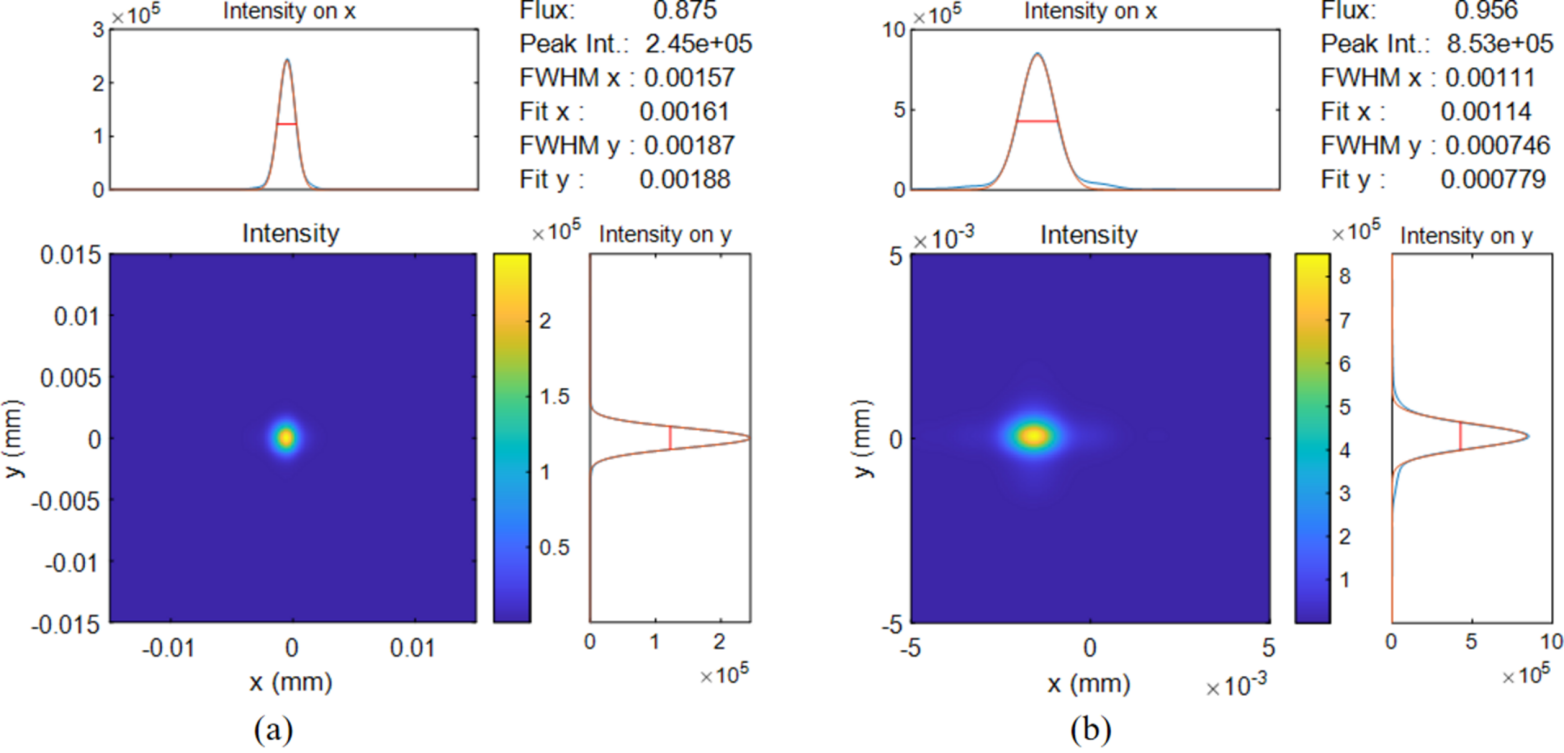
Focusing spot at the sample by the optimal mirror design for (*a*) 400 eV and (*b*) 900 eV.

Compared with the traditional full-length cooling method, the optical tracing simulation shows that the multi-segment cooling design of mirror M1 significantly improved the beam’s focusing performance of X-rays at the sample position, as shown in Fig. 7 ▸. The stray light within the focus spot at the sample point was successfully eliminated at the photon energy of 900 eV, and a significant improvement in focus quality was observed at the photon energy of 400 eV.

Simultaneously, the intensity of the focus spot at the sample point was compared for conditions of full-length cooling and multi-segment cooling, as shown in Figs. 3 ▸ and 7 ▸. For photon energy of 900 eV, the stray light of the focus spot in the full-length design was successfully eliminated by the multi-segment cooling design, and the peak value of the focus spot intensity was significantly enhanced by 176.9%, from 3.08 × 10^5^ to 8.53 × 10^5^. For the photon energy of 400 eV, as the focusing effect of the spot was enhanced, the intensity of the focused spot also showed a significant improvement, by 58.1%, from 1.55 × 10^5^ to 2.45 × 10^5^.

Additionally, given that the heat load at other energy points is more than 50% lower than that at the 400 eV and 900 eV energy points, and considering that the FEL-II beamline prioritizes 400 eV and 900 eV for critical applications, while other energy points are seldom utilized, the mirror’s optimization has been primarily focused on these two key energy points. Nonetheless, the optimized mirror design has also been evaluated for its focusing performance at other energy points, and it has satisfied the requirements of optical performance within other cases.

Consequently, the optimization of the mirror’s cooling length and the positioning of its cooling grooves has effectively controlled the thermal deformation under high heat loads. This multi-segment cooling design of the high-heat-load reflection mirror has resulted in a successful enhancement of the beam’s focus spot quality at the sample point in the FEL-II beamline of the SHINE facility.

## 5. Conclusion

This paper presents a new method for managing thermal deformation of high-heat-load mirrors in XFEL beamlines. The cooling length and position of cooling grooves plays a significant role in the management of the mirror’s thermal deformation under high heat load and in the improvement of the beam’s focus spot at the sample.

Optimizing the mirror’s cooling configuration and the precise placement of the cooling groove has substantially augmented the efficacy of X-ray transmission from the light source to the endstation’s sample, which is important for the endstation of the beamline. The multi-segment cooling design of the first reflection mirror will ensure stable operation of the SHINE facility.

## Figures and Tables

**Table 1:** Physical properties of the cooling water

Density (kg m^−3^)	Flow rate (m s^−1^)	Specific heat capacity (J kg^−1^ °C^−1^)	Viscosity (Pa s)	Inner diameter of tube (mm)	Thermal conductivity (W m^−1^ °C^−1^)
1000	0.5	4200	0.8 × 10^−3^	8	0.62

## Data Availability

Data will be made available on request.
